# A temporal window for estimating surface brightness in the Craik-O'Brien-Cornsweet effect

**DOI:** 10.3389/fnhum.2014.00855

**Published:** 2014-11-03

**Authors:** Ayako Masuda, Junji Watanabe, Masahiko Terao, Akihiro Yagi, Kazushi Maruya

**Affiliations:** ^1^Department of Integrated Psychological Science, Kwansei Gakuin UniversityNishinomiya, Japan; ^2^NTT Communication Science Laboratories, Nippon Telegraph and Telephone CorporationAtsugi, Japan; ^3^Department of Life Sciences, University of TokyoMeguro, Japan

**Keywords:** Craik-O'Brien-Cornsweet effect, brightness induction, temporal window, masking, subjective contour

## Abstract

The central edge of an opposing pair of luminance gradients (COC edge) makes adjoining regions with identical luminance appear to be different. This brightness illusion, called the Craik-O'Brien-Cornsweet effect (COCe), can be explained by low-level spatial filtering mechanisms (Dakin and Bex, 2003). Also, the COCe is greatly reduced when the stimulus lacks a frame element surrounding the COC edge (Purves et al., 1999). This indicates that the COCe can be modulated by extra contextual cues that are related to ideas about lighting priors. In this study, we examined whether processing for contextual modulation could be independent of the main COCe processing mediated by the filtering mechanism. We displayed the COC edge and frame element at physically different times. Then, while varying the onset asynchrony between them and changing the luminance contrast of the frame element, we measured the size of the COCe. We found that the COCe was observed in the temporal range of around 600–800 ms centered at the 0 ms (from around −400 to 400 ms in stimulus onset asynchrony), which was much larger than the range of typical visual persistency. More importantly, this temporal range did not change significantly regardless of differences in the luminance contrast of the frame element (5–100%), in the durations of COC edge and/or the frame element (50 or 200 ms), in the display condition (interocular or binocular), and in the type of lines constituting the frame element (solid or illusory lines). Results suggest that the visual system can bind the COC edge and frame element with a temporal window of ~1 s to estimate surface brightness. Information from the basic filtering mechanism and information of contextual cue are separately processed and are linked afterwards.

## Introduction

Brightness induction is a phenomenon in which the estimated brightness of a region of space is influenced by the spatio-temporal luminance pattern of surrounding regions (e.g., Bloch, 1885; Heinemann, 1955; De Valois et al., 1986; Eagleman et al., 2004). Low-level spatial filtering mechanisms have been shown to play a major role in the brightness illusion (Blakeslee and McCourt, 1997, 1999, 2001, 2004; Dakin and Bex, 2003; Blakeslee et al., 2008). In the Dakin and Bex (2003) model, visual images rendered in high-spatial-frequency channels still contain some low-spatial-frequency content. The visual system enhances the gain of those residual low-frequency components so that they are near normal levels, creating illusory brightness effects. They showed this model can explain a type of brightness induction phenomenon. Also, a series of papers by Blakeslee and McCourt showed that their model, called the ODOG (oriented difference-of-Gaussians), can explain most of the brightness illusion, including phenomena that had been considered to be higher-order effects (Blakeslee and McCourt, 2012). The core idea of the ODOG model could be stated as follows: Despite the fact that the ODOG model possesses filters tuned to very low spatial frequencies, because the range of filter frequencies is finite there will inevitably be some low spatial frequency information that is lost. The reconstituted image will therefore be missing some of its original low frequency components. When the low frequency components of an image are subtracted from it this causes induction.

**Figure 1:**
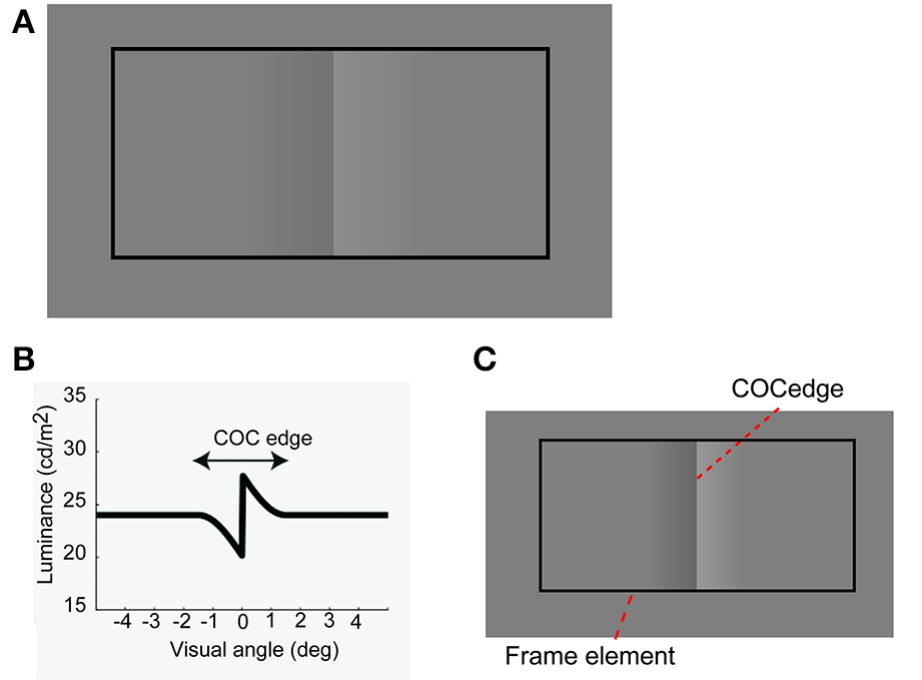
**The Craik-O'Brien-Cornsweet effect. (A)** Stimulus and phenomenon. **(B)** Luminance profile of an opposing pair of luminance gradients at the center. **(C)** Diagram of COCe stimulus.

The Craik-O'Brien-Cornsweet effect (COCe; Figure 1A, O'Brien, 1958; Craik, 1966; Cornsweet, 1970) is a brightness induction phenomenon in which the central edge of an opposing pair of luminance gradients (COC edge, Figures 1B,C) makes adjoining regions with identical luminance appear to have different luminance. This effect can be basically explained by a low-level filtering mechanism (Dakin and Bex, 2003). In addition, recent brain imaging and physiological studies have shown that the early visual cortical areas, starting as early as the primary visual cortex, are activated when the COCe is observed (e.g., Roe et al., 2005; Boyaci et al., 2007; Hung et al., 2007; for reviews, see von der Heydt et al., 2003; Komatsu, 2006). However, the COCe can also be affected by contextual cues, such as the lighting direction in the environment (Purves et al., 1999). For example, Purves et al. (1999) elegantly demonstrated that the COCe decreases considerably when there is no frame element surrounding the COC edge and the COC edge is drawn on a uniform background. When the extra contextual cue, i.e., a frame element, are provided to indicate the lighting direction and uniformity, the brain then interprets the surfaces as having different surface reflectances. Given that a filtering mechanism can explain a majority of the COCe, we hypothesize that information from the basic filtering mechanism and information of contextual cue are separately processed and are bound afterwards.

In this study, we investigated whether contextual cues can be dissociated from the basic filtering mechanism. We examined this question by focusing on the temporal characteristics of COCe. Blakeslee and McCourt (2008) showed that the brightness induction can occur almost immediately after the stimulus presentation. These results indicate that is the processing basic filtering mechanism is very rapid. This fast processing of COCe is probably supported by the basic filtering mechanism. Although the speed of contextual cue processing is unclear, it might be slow since it might include the steps of recognition and integration of image features across the image. If any speed difference exists between the basic filtering mechanism and the contextual cue processing, visual system should hold information from fast filtering mechanism to bind information from the slow contextual cue processing. This means that visual system would have a temporal window to bind two types of information. In the conventional COCe display, the frame element and COC edge are tightly linked to each other because they are displayed at the same time. This would make it hard to assess the presence of any dissociation and binding. In this study, we therefore introduced a physical time difference between the frame element and COC edge to examine the possibility of the dissociation and binding. If the COCe and contextual effect are processed by means of a single fast mechanism, the COCe could not be modulated when the frame element and the COC edge are presented temporally apart from each other. In contrast, if the contextual cue effect can be somehow separated from the basic COCe processing and then linked to the COC-edge information, context modulation might be observed even when the frame and COC edge are displayed asynchronously. Several studies on cross-modal integration have shown that contextual cross-modal integration, e.g., the McGurk-MacDonald illusion (McGurk and MacDonald, 1976), can occur even when audio-visual stimuli are desynchronized to several 100 ms (Soto-Faraco and Alsius, 2007, 2009; Kitagawa and Kitamura, 2014). Although such a cross-modal modulation effect appears to be caused in an integration stage higher than that where the COCe is caused, it is conceivable that a contextual cue could also be effective for the COCe when uni-modal stimuli are desynchronized to several 100 ms.

**Figure 2:**
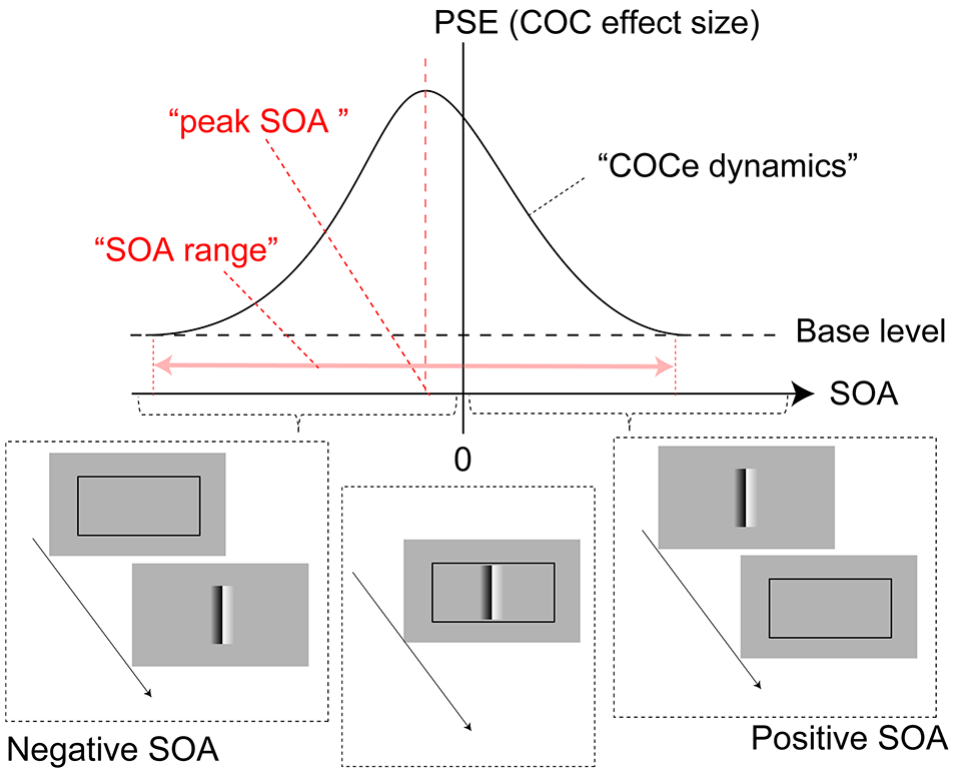
**Diagram of COCe dynamics**. The horizontal dotted line shows a base level PSE measured in the control condition where no frame element was displayed. The red arrow shows the range of SOA in which the presence of the frame element enhances the COCe.

We investigated the temporal range where the contextual cue affects the COCe (Figure 2). We measured the size of the COCe while varying the onset asynchrony between the COC edge and frame element [Hereafter, we call this difference the stimulus onset asynchrony (SOA)]. The results showed that the width of the temporal range in relating the surface information and local luminance fluctuation signals for the COCe could be large (>600 ms), exceeding the typical length of visual persistency (up to a few 100 ms), and constant with various types of frame elements.

## Materials and methods

The methods were basically the same in all experiments unless stated otherwise in the method section for each experiment.

### Observers

Three observers participated, one of the authors and two others who were volunteers and unaware of the purpose of the experiments. All observers had normal or corrected-to-normal vision. The dominant eye was determined for each observer by the Dolman method (Fink, 1938). Informed consent was obtained from all participants before the experiment. Recruitment of the participants and experimental procedures were conducted in accordance with the Declaration of Helsinki.

### Apparatus

Stimuli were generated using a PC with a Psychlops library (http://psychlops.sourceforge.jp/en/) and displayed on a 21-inch CRT monitor (TOTOKU Calix CDT2141A) with a refresh rate of 100 Hz. An 8-bit grayscale with gamma correction was provided by a video card (Aopen GeForce4Ti4200 with AGP8X). The observer viewed the monitor from a distance of 64 cm while sitting in a completely dark room with his/her head fixed on a chin rest. The spatial resolution of the monitor was 1280 × 1024 pixels, with each pixel subtending 1.6 min at the viewing distance of 64 cm. The stimuli were presented at the center of the monitor, and the observers viewed the stimuli with both eyes.

### Stimuli

**Figure 3:**
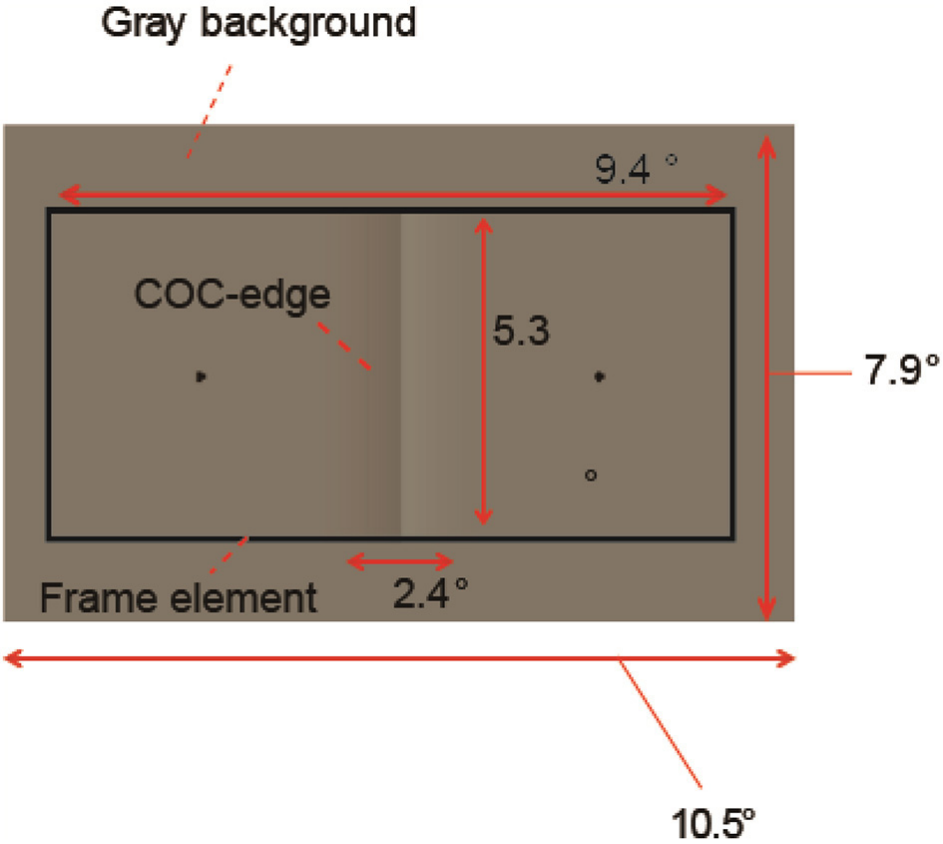
**Stimulus configuration**.

The stimulus was drawn on a gray background consisting of two flanking gray squares. The size of the gray background was 10.5 (width) × 7.9 (height) deg (Figure 3). The luminance of the two squares was varied from 23.8 cd/m^2^ and the contrast between them (surface contrast) was varied in eight steps (from −8 to 32% for observer YM and TF) or 10 steps (−24–32% for observer AM). The negative values denote that luminance of the right square was high and vice versa. The COC stimulus consisted of two major components. One is the COC edge displayed at the center of the gray background (Figure 1C). The size of the COC edge was 2.4 (width) × 5.3 (height) deg. As in the standard COC stimulus, the luminance was abruptly changed along the vertical centerline and gradually changed toward the gray level as the distance from the centerline increased (Figure 1B). The centerline was made by adjacent rows of dark (19.9 cd/m^2^) and bright (27.6 cd/m^2^) pixels. This value corresponds to 16% as the Michelson contrast. From the center to the right end of the COC edge, the luminance was gradually changed from 27.6 cd/m^2^ to the same luminance level as for the right side of the gray background. Similarly, the luminance was gradually changed from 19.9 cd/m^2^ to the luminance level at the leftmost of the gray background from the center to the left end. The other element was the rectangular frame element (Figure 1C). The frame element was drawn with thin solid lines. The width of the thin line was 0.2°. The size of the frame element was 9.4 (width) × 5.3 (height) deg and the frame element was centered at the gray background. The contrast of the frame element against the mean luminance of the gray background (23.7 cd/m^2^) was varied in five steps from 5 to 100%. Small black (0.01 cd/m^2^) dots were also displayed at 3.6° left and right of the center of the gray rectangle (probe elements). Observers were asked to compare the lightness around these two locations.

### Data analysis

**Figure 4:**
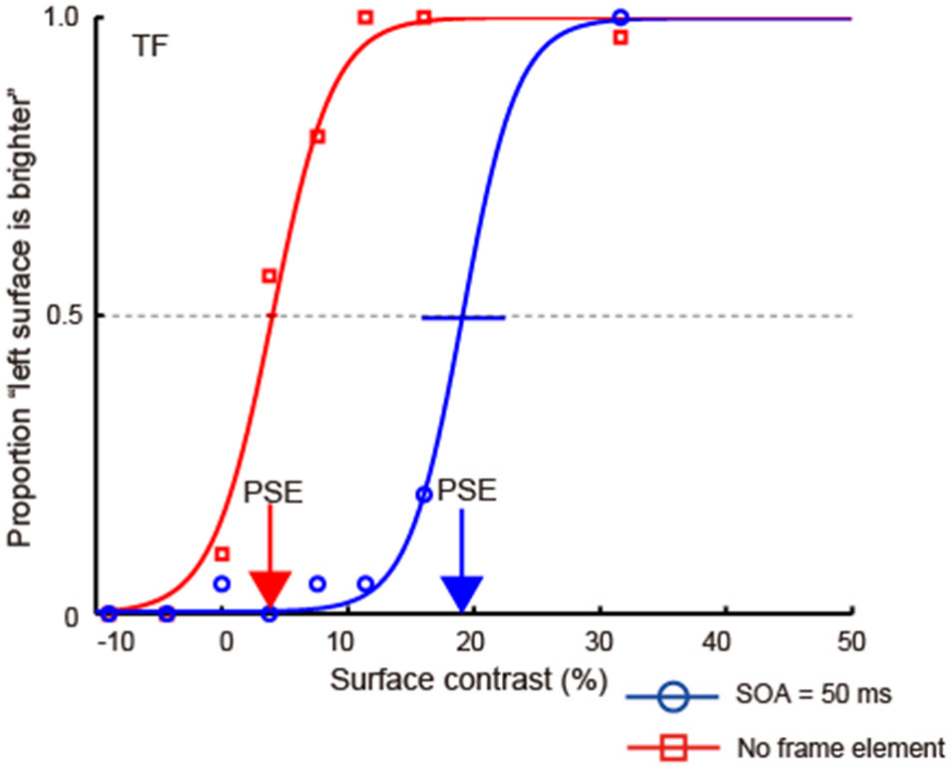
**An example of psychometric functions from experiment 1**. In this condition, the contrast of the frame element was 100% and the duration of the frame element and COCe edge was 50 ms. Each point plots the proportion of times observers responded “left surface is brighter” (y-axis) against the luminance contrast of the surfaces (x-axis). The negative values on the x-axis denote that luminance of the right surface was high. Cumulative Gaussian functions were then fitted to these data (solid lines). Error bars indicate 95% confidence intervals computed by bootstrapping. Red and blue arrows indicate the point of PSE for each condition.

We plotted the proportion of times the observers reported that the left region was brighter as a function of the physical luminance contrast and then fit a cumulative Gaussian psychometric function (Figure 4). We used the Psignifit Toolbox Version 2.5.6 in Matlab (Wichmann and Hill, 2001) for fitting. We estimated the surface luminance contrast where the performance became 50% for each observer and condition, i.e., the point of subjective equality (PSE). The PSE value is the physical luminance contrast that cancels the brightness induction caused by the luminance gradient. As the COCe becomes stronger, the PSE shifts to the positive direction. Thus, the PSE can be used as an index of the strength of the COCe. We also computed the 95% confidence interval by bootstrapping. The bootstrap ran 30,000 times.

## Experiment 1

### Methods

In this experiment, a frame element consisting of solid black lines and a COC edge were presented. Before the initiation of a trial, three fixation targets (white crosses 0.5 × 0.5° in size and 78.0 cd/m^2^ in luminance) were displayed at the center of the stimulus field and 6.3° above and below it. When observers pressed a button, the stimulus sequence started. The COC edge and frame element were displayed at physically different times. The gray background and probe elements were displayed continuously, and the frame element and COC edge were displayed with given durations and SOA. The SOA for the frame element and COC edge was varied in 15 steps from −400 to 400 ms. Here, the sign of the SOA indicates the display order of the frame and COC edge components, and positive SOA indicates that the onset of the COC edge precedes the onset of the frame element (Figure 2). The duration of the COC edge and frame element was the same. The durations were 50 and 200 ms, and durations of these two lengths were run in separate blocks. The Michelson contrast of the frame element was varied in five steps (5, 10, 20, 50, and 100%). After a whole sequence was displayed, a uniform black field was displayed and observers made a response by a button press. Observers were asked to judge which region of the area around the left and right probes was lighter.

The experiment was conducted in sessions. Within a session, the contrast and duration of the frame element were fixed. In a session, the surface contrast was varied, while the polarity of the COC edge was fixed so that the right flanking region was perceived lighter when the COCe occurs. Twenty or thirty (for observer AM) trials were repeated for each contrast condition of the gray background in randomized order. As a control, we asked observers to perform the same task in the condition where only the COC edge was displayed. In this control condition, only the COC edge was displayed for 200 ms, and observers judged which region of the area around the left and right probes was lighter. The net trial numbers were 12,160 for observer YN and TF (8 surface contrasts × 5 frame contrasts × 15 SOAs × 20 repetitions + control condition: 8 surface contrasts × 20 repetitions) and 22,800 for observer AM (10 surface contrasts × 5 frame contrasts × 15 SOAs × 30 repetitions + control condition: 10 surface contrasts × 30 repetitions), taking ~100 h, including rest periods.

### Results

Figure 4 shows an example of psychometric functions from an observer (TF). In general, when the surface contrast was positive (the luminance of the right region was low), the observers reported that they perceived the left region as being lighter, indicating that the observers could judge the surface brightness properly. When both the frame and COC edge were displayed, the psychometric functions were shifted in the direction of positive surface contrast. In the control condition, where no frame element was displayed, the shift decreased. Thus, the COCe was attenuated severely without the frame element.

**Figure 5:**
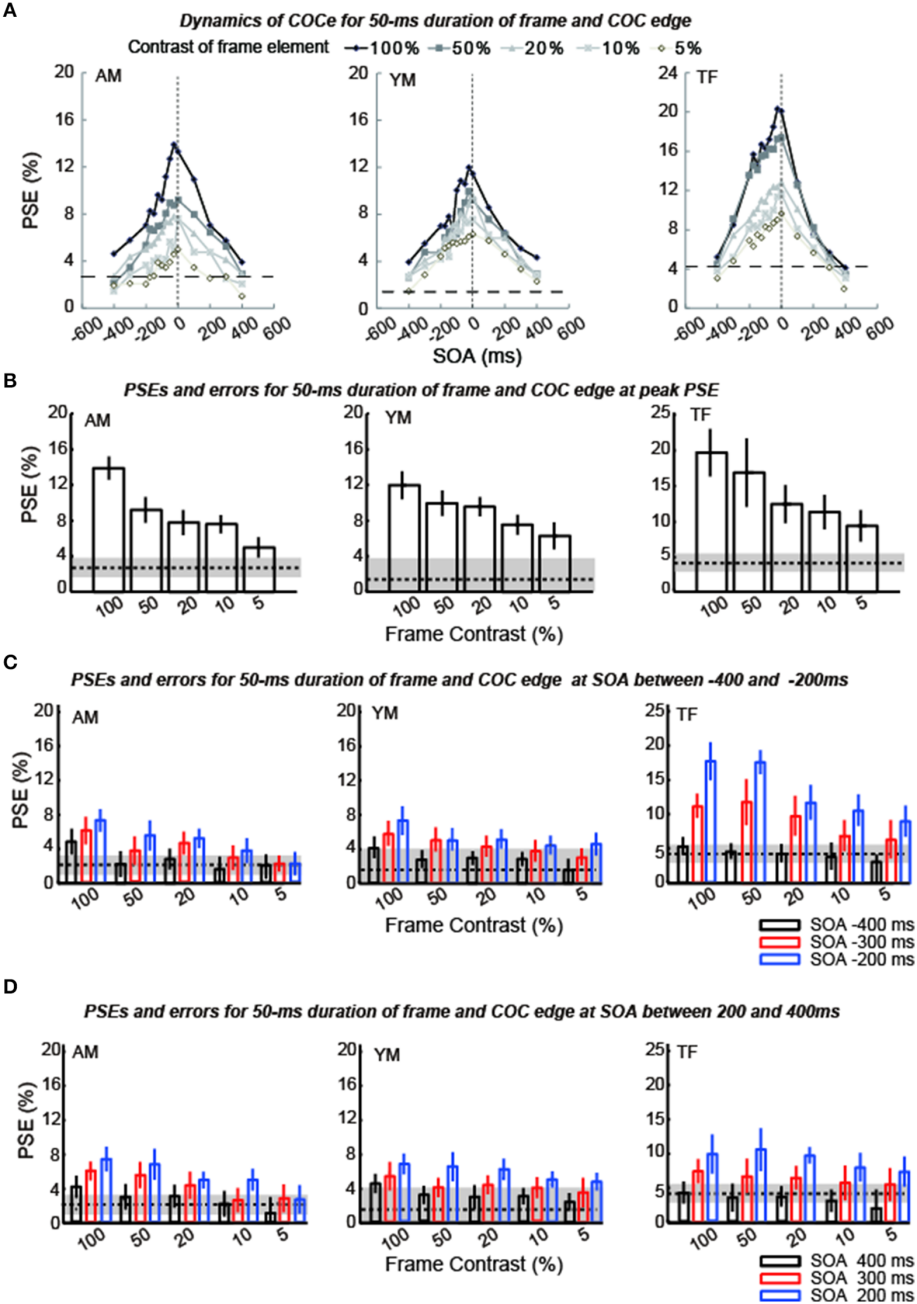
**Results of experiment 1 (durations of COC edge and frame element were 50 ms). (A)** Estimated PSEs for each observer are plotted as functions of SOA between the frame element and COC edge. Vertical dotted lines show that the SOA is zero. Horizontal thick dotted lines show the PSE estimated for the control condition in which the frame element was not shown. The difference between each line and symbol indicates the contrast of the frame element (5–100%). **(B)** Estimated PSEs and errors at peak estimated point in **(A)** for each frame contrast. Error bars indicates 95% confidence intervals computed by bootstrapping. Horizontal dotted lines indicate PSEs estimated for the control condition where the frame element was not shown. The shaded region indicates 95% confidence intervals in the control condition. **(C)** Estimated PSEs and errors at −400 ms SOA, −300 ms SOA, and −200 ms SOA shown in **(A)** for each frame contrast. **(D)** Estimated PSEs and errors at 400 ms SOA, 300 ms SOA, and 200 ms SOA shown in **(A)** for each frame contrast.

**Figure 6:**
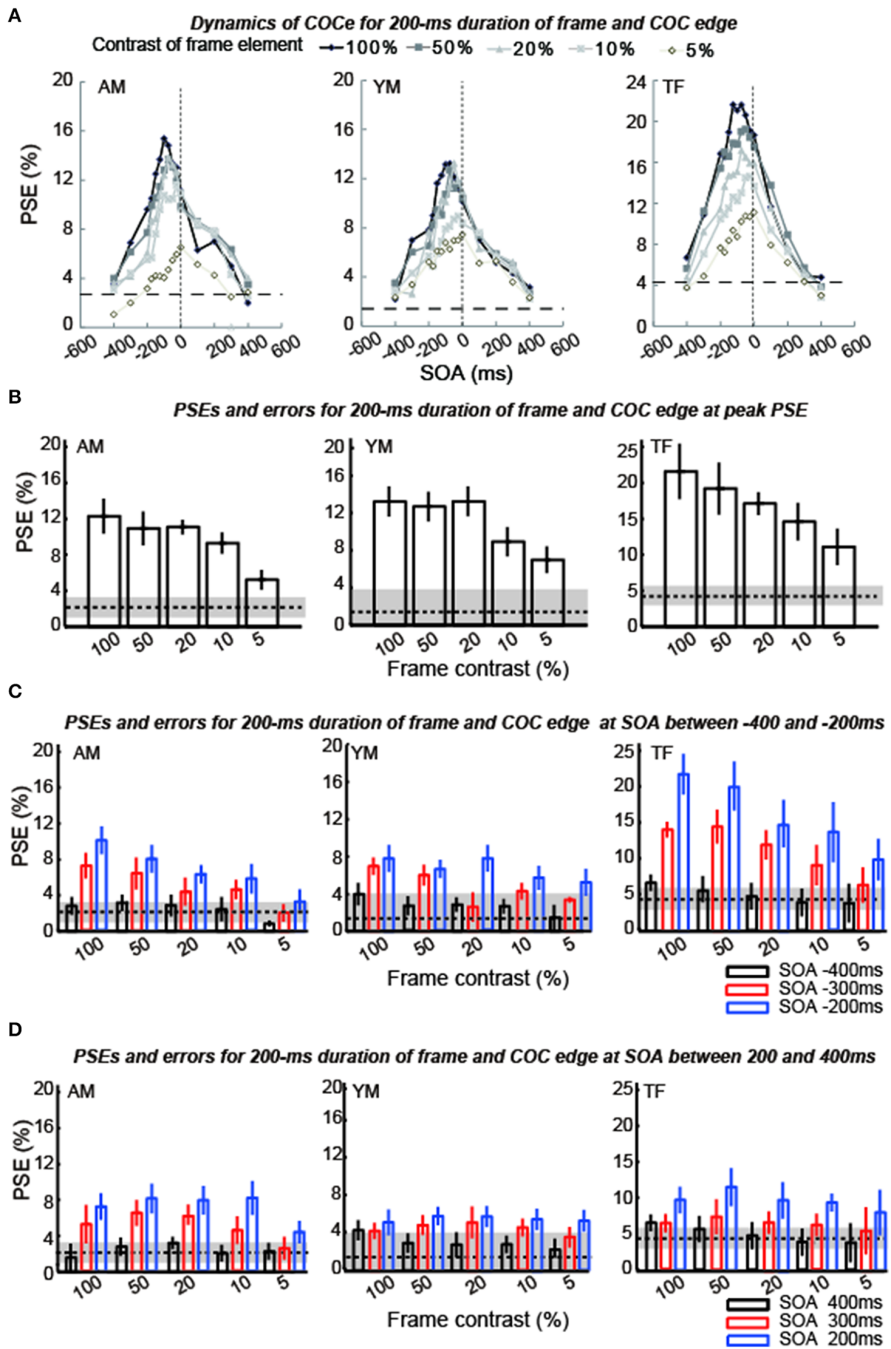
**Results of experiment 1 (durations of COC edge and frame element were 200 ms)**. The description of each graph is the same as in the caption of Figure 5.

The apparent brightness was estimated for each condition by calculating the PSEs. When the COCe is observed, the PSE is yielded at a positive contrast value, and a larger PSE indicates that a stronger COCe occurred. In all conditions, the dynamics of the PSE against SOA conditions (COCe dynamics) draws a bell-shape (Figures 5A, 6A for 50 ms durations of the COC edge and frame element and for 200 ms durations, respectively). The maximum PSE was around 15% (15.4, 13.2, and 21.6% for AM, YM, and TF, respectively). In the control condition, the estimated PSEs were around 3% (2.7, 1.4, and 4.2% for AM, YM, and TF, respectively). The maximum COCe size increased as the luminance contrast of the frame element increased when the durations of COC edge and frame element were 50 ms (Figure 5B) and 200 ms (Figure 6B). The COCe decreased as the absolute value of SOA increased. The COCe at largest SOAs (±400 ms) was not significantly different from the COCe in the control condition among all contrast and duration conditions (Figures 5C,D, 6C,D). In most conditions, the range of the SOA, in which the presence of the frame element was significantly effective (hereafter called the SOA range; see also Figure 2) was between −300 and 300 ms, except for several conditions where the frame duration was 50 ms and the contrast was low (< = 10%). Thus, the observed width of the SOA range was typically 600–800 ms, roughly consistent with contrast and duration change, yet some exceptions were observed.

The COCe at the peak SOA increased as the frame contrast increased for both the 50- and 200 ms frame and COC edge durations. More interestingly, only in the 200 ms duration condition, the peak SOA was shifted in the negative SOA direction when the frame contrast increased. The negative sign of SOA denotes the frame element preceded the COC edge. When the frame contrast was 5% and the durations of frame and COC-edge were 200 ms, the peak SOA was at 0 ms. When the frame contrast was 100%, it was at around −100 ms. In contrast, when the duration was 50 ms, such negative shift of peak SOA was not observed. The peak SOA was at around 0 ms irrespective of the frame contrast.

### COCe size was not affected by the duration change of COC edge

**Figure 7:**
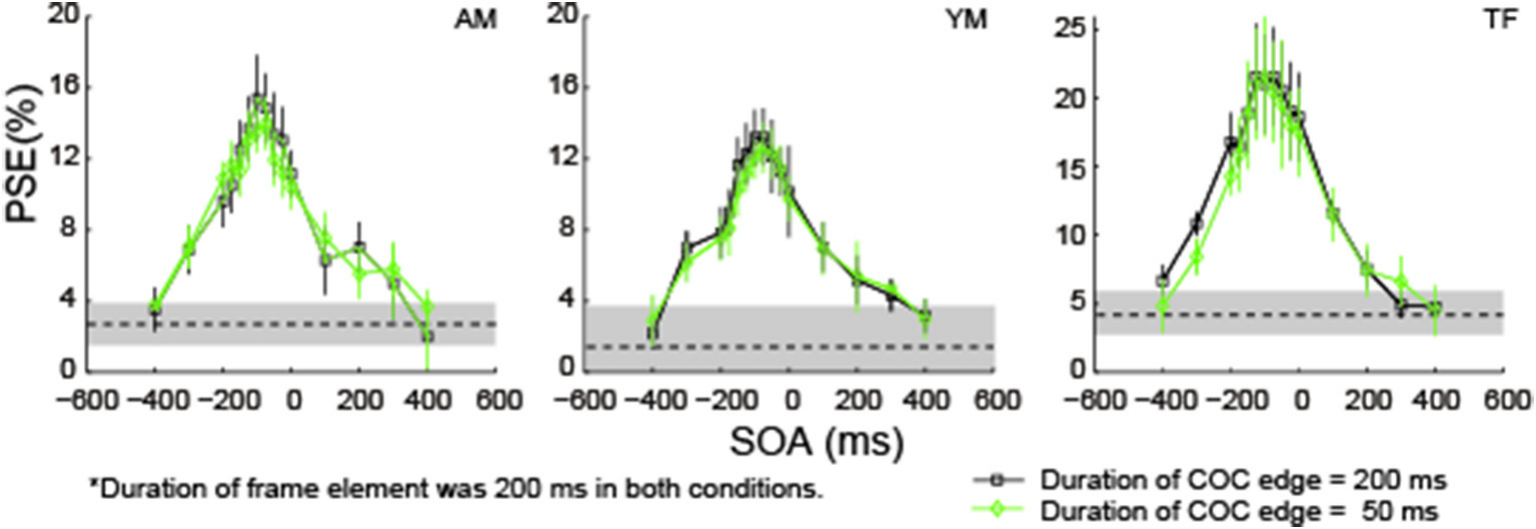
**The COCe while only the COC edge duration was varied**. Estimated PSEs for each observer are shown. Error bars indicate 95% confidence intervals computed by bootstrapping. Horizontal dotted lines show the PSE in the control condition where the frame element was not shown. The shaded region indicates 95% confidence intervals in the control condition.

In the first experiment, the duration of the COC edge was the same as that of the frame element. When the duration of the frame element was varied with a fixed SOA, both the period when the COC edge and frame element were superimposed and the inter-stimulus interval (ISI) between them are co-varied. The question here is whether those variations affected the COCe dynamics. To answer it, we fixed the duration of the frame element at 200 ms and compared COCe dynamics between conditions where the durations of the COC edge were 50 and 200 ms. The observers and methods were the same as in the first experiment except for the durations of the COC edge and frame element. Results show that the PSEs were virtually the same, irrespective of the duration of the COC edge (Figure 7). This indicates that the COCe size does not depend on the duration of the COC edge or the period in which both the COC edge and frame element are displayed simultaneously.

### Interocular presentation

Next, we examined whether the COCe dynamics is affected by the interocular presentation. When observers view the COC edge and frame element with different eyes, full information about the COCe is presumed to be available only after binocular fusion. If the stage where the contextual modulation by the frame element occurred after binocular fusion, the delay should not affect the COCe dynamics.

The stimulus and procedure were the same as in the first experiment except for the following modifications. The display area of the monitor was horizontally divided into two areas and the observers viewed two stimuli presented in each area through a mirror stereoscope so that each eye could see its corresponding stimuli. Observers viewed the frame element and COC edge with different eyes through the mirror stereoscope. The optical distance from the monitor to the observer's eye was 64 cm. In the interocular display condition, observers viewed the frame elements with the left eye and viewed the COC edge with the right eye. Observers viewed other elements with both eyes. We also tested a monocular viewing condition, in which observers viewed all elements with only their right eye. The SOA was varied in nine steps from −400 to 400 ms. The duration of the frame stimulus and COC stimulus was fixed at 200 ms. For all observers, the surface contrast was varied in 10 steps from −24 to 32%, where negative values denote that the luminance of the right square was high and vice versa.

**Figure 8:**
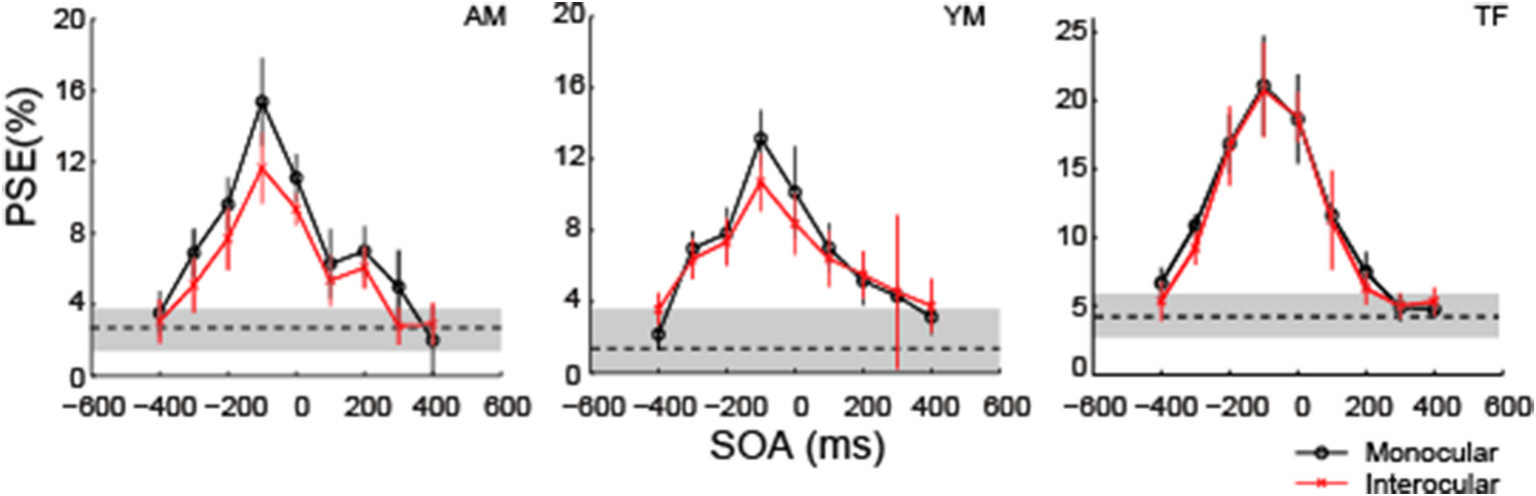
**The COCe with monocular and interocular displays**. Estimated PSEs from each observer are shown. Error bars indicate 95% confidence intervals computed by bootstrapping. Horizontal dotted lines show the PSE for the control condition where the frame element was not shown. The shaded region indicates 95% confidence intervals in the control condition.

Figure 8 shows the results in the monocular viewing and interocular display conditions. The COCe dynamics was virtually the same between the monocular and interocular conditions, yet the observed COCe was slightly smaller in the latter. Peak SOAs were around −100 ms for both conditions. This is consistent with the results of the 200 ms duration condition in the first experiment. These results show that the COCe dynamics is not affected by the interocular presentation. This indicates that the main stage of the COCe processing lies after the binocular fusion (Masuda et al., 2011).

## Experiment 2

**Figure 9:**
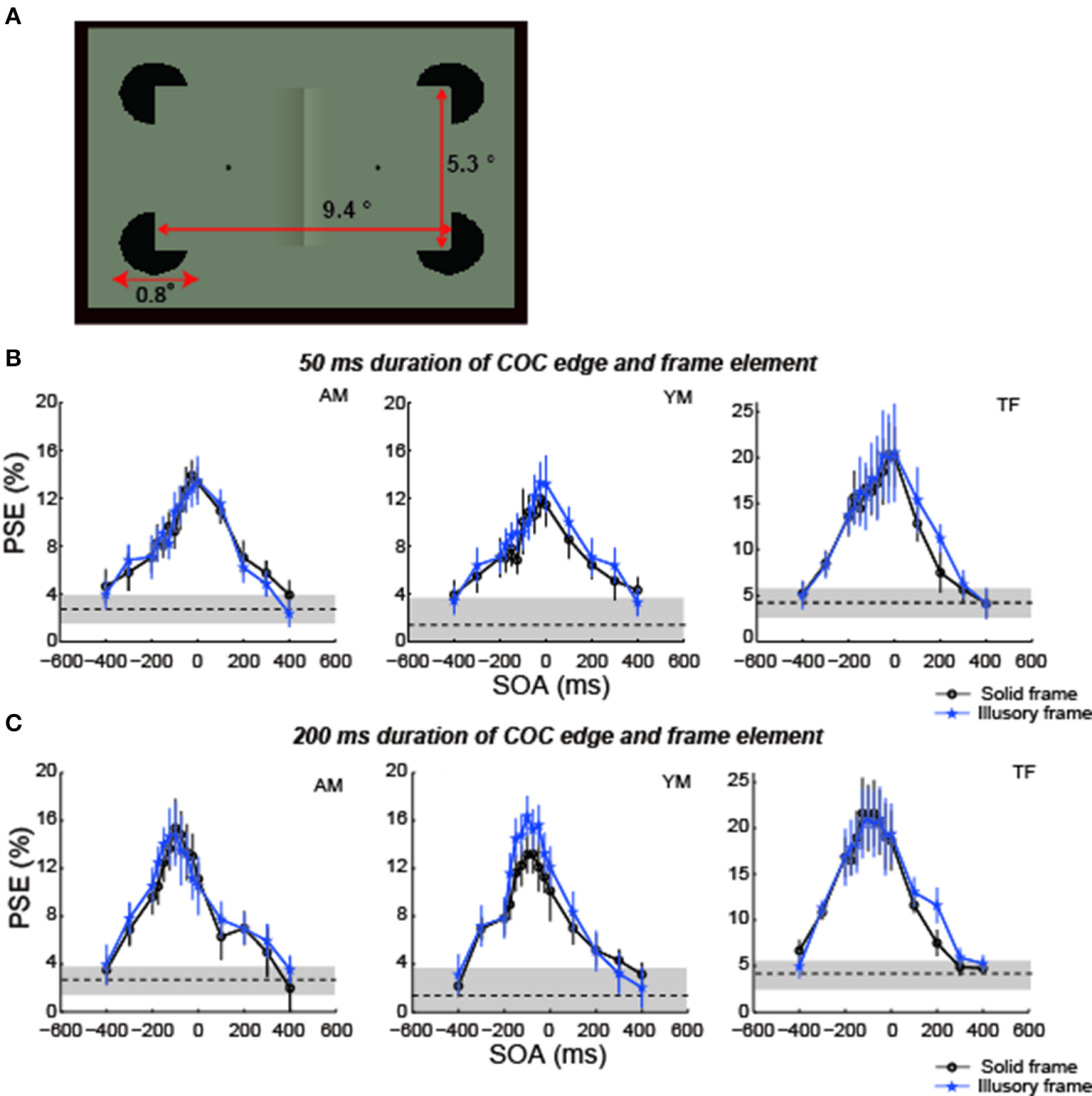
**The COCe with an illusory frame element. (A)** Diagram of stimulus. **(B)** Results for 50 ms duration. **(C)** Results for 200 ms duration. Data shown in black are replotted from the 100% contrast and 200 ms duration condition in experiment 1. Error bars indicate 95% confidence intervals computed by bootstrapping. Horizontal dotted lines show the PSE estimated for the control condition where the frame element was not shown. The shaded region indicates 95% confidence intervals in the control condition.

It is well known that figure-ground segregation occurs not only for solid lines but also for illusory lines (Kanizsa, 1955). For example, an illusory rectangle is perceived when we see four sectored disks aligned as shown in Figure 9A. The COCe occurs not only with a frame element consisting of solid lines but also with subjective rectangles consisting of such illusory lines. It has been reported that the temporal aspect of the illusory contour is different from the real contour. For example, Lee and Nguyen (2001) reported that the onset of monkey V1 and V2 neuronal responses to illusory contours occur about 30–50 ms later than to solid contours. The processing time for illusory contours would be different from, probably longer than, that for real contours. One intriguing question is whether this difference in the temporal aspect affects the dynamics of the COCe. In this experiment, we measured the COCe dynamics with a frame element consisting of illusory lines induced by four sectored disks.

### Methods

Three observers, including one of the authors, participated in this experiment. All three observers participated in experiment 1. The stimulus and procedures were basically the same as in experiment 1 except that in this experiment the frame element consisting of illusory contours was presented by four black (0.01 cd/m^2^) sectored disks. The diameter of the sectored disks was 0.8°, and the sectored angle was 90°. The net trial number was 2400 for observers YN and TF (8 surface contrasts × 15 SOAs × 20 repetitions) and 4500 for observer AM (10 surface contrasts × 15 SOAs × 30 repetitions), taking ~10 h, including rest periods.

### Results

Compared with the PSEs estimated in the condition using a frame element of 100% contrast in experiment 1, the COC dynamics and the peak SOA with illusory frame elements were virtually the same as those observed with solid frame elements in both conditions with 50 ms durations of the COC edge and frame element and with 200 ms durations (Figures 9B,C). These results show that differences in the temporal aspect between real and illusory contour processing do not affect the temporal aspect of the COCe.

## General discussion

In this study, we investigated whether contextual cues can be dissociated from the filtering mechanism. For this purpose, we examined whether the COCe is modulated by a contextual cue displayed before or after the main component. We measured the size of the COCe while varying the SOA between the presentations of the COC edge and frame element. We found that the COCe was modulated even when the frame element and COC edge was displayed at the widely different times. The range of SOA where the COCe was larger than the baseline COCe, which was observed when the stimulus consisted of only the COCe edge, was roughly constant (±300–400 ms) among the various frame display conditions (stimulus type, presentation eye, duration, and contrast). Also, in several conditions where the duration of frame element was relatively long and its contrast was high, the SOAs that gives a maximum COCe (peak SOA) shifted in the direction of negative SOA. For example, the peak SOA was at ~100 ms when the frame contrast was 100% and the duration of frame element and COC edge was 200 ms. These results are consistent with a view that the contextual cue effect can be, at least partially, separated from the basic COCe processing.

The estimated length of the time window, greater than 600 ms, is fairly large as a length of a perceptual integration window. Typically, elements should be displayed within a window of up to 200 ms to be fused in the observer's subjective view. For example, it is well known that a briefly presented stimulus remains visible for a short period after the stimulus offset. The typical duration of this phenomenon, called “visual persistency,” is 150–200 ms from the onset of the stimulus (Efron, 1970; Di Lollo, 1977; Coltheart, 1980). We admit that the COC edge and frame element would be fused into one mental image within some temporal range. However, considering the long temporal window observed here, it is unlikely that visual persistency can explain the whole pattern of the results. Even in the conditions where the SOA between those elements was ±200–300 ms, which exceeds the limit for visual persistency, a modulation of the COCe by the contextual cue was observed to some extent.

The measured SOA range did not change between solid and illusory frame conditions. In addition, it was not influenced by the interocular presentation of the frame and COC edge. These results show that the lower limit of contextual-cue processing lies in the early visual areas in which binocular fusion begins. Many physiological studies have reported that V1 and V2 neurons of non-human primates are responsive to illusory contours (von der Heydt et al., 1984; Lee and Nguyen, 2001; Ramsden et al., 2001; Seghier and Vuilleumier, 2006).

The dissociation of contextual COCe modulation from the main filtering mechanism shown in this study does not necessarily indicate that the processing of contextual modulation is mediated by the high-level cognitive mechanism. One may suspect that the long temporal window observed in this study indicates that the contextual modulation occurs at a level that requires several steps after the low-level spatial filtering. The contextual effect in an audio-visual stimulus, like the McGurk-MacDonald illusion, typically shows an integration window of over ±300 ms. This quantitative similarity in the integration temporal window between multimodal studies and present studies might appear to support the view that the contextual modulation in the COCe is also mediated by the high-level mechanism. Considering the presence of the sustained channel in the human visual system (Kulikowski and Tolhurst, 1973; Tolhurst, 1973, 1975), however, the early mechanism, not only the high-level system, is able to hold the preceding information, and the long temporal window does not necessarily indicate high-level processing.

**Figure 10:**
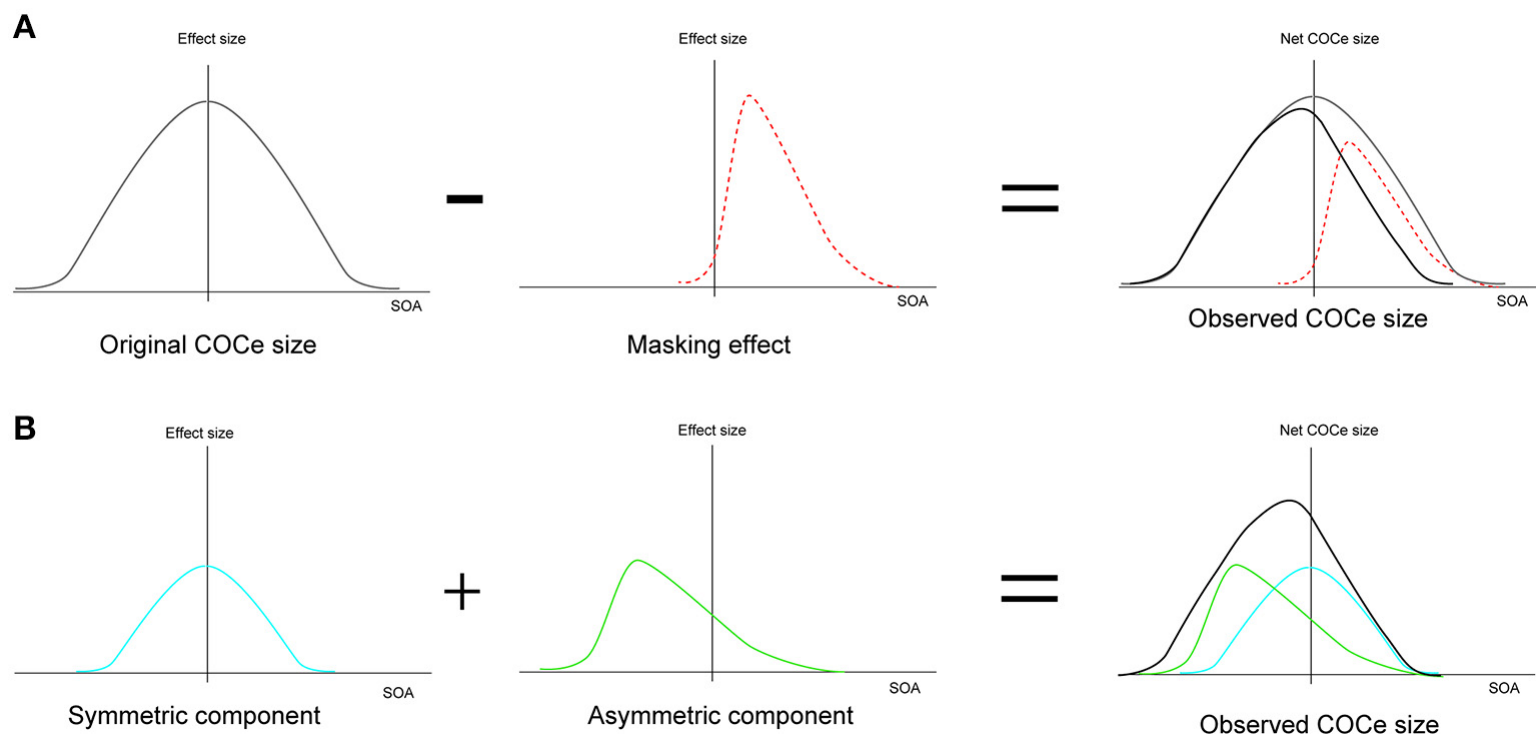
**Two explanations for the negative shift of peak SOA. (A)** Explanation by metacontrast masking. **(B)** Explanation by two mechanisms located at different processing levels.

The low-level filtering mechanism is supposed to be very rapid (Blakeslee and McCourt, 2008). Considering that a high-level effect of this sort might be slow since it would require the steps of recognition and integration of image features across the image, it might take some time for information about a contextual cue to influence the strength of the observed brightness even when all of the information is concurrently given on the retina. This means that a processing time difference could emerge between a contextual cue and the basic COCe. The peak SOA might reflect the time difference between rapid COCe processing and contextual-cue processing more directly. The present results show a negative shift of peak SOA in some conditions. As we described in the introduction, it might take time for the contextual information, i.e., the display of the frame element, to influence the COCe. However, at present, we cannot conclude that this negative shift is clear evidence supporting high-level processing of contextual cues, because the negative shift can be explained in several ways, including by a low-level mechanism. For example, it might reflect temporally asymmetric decreases in the effective contrast of the COC edge and in the effect size in the low-level filtering mechanism due to asymmetric metacontrast masking by the frame element (Figure 10A, Stigler, 1910; Alpern, 1953; Kahneman, 1967; Breitmeyer, 1984; Enns and Di Lollo, 2000; Breitmeyer and Ogmen, 2006). However, the results of previous studies on metacontrast masking—the influence of frame contrast (cf. Breitmeyer and Ogmen, 2006) or the effect of interocular viewing (cf. Schiller and Smith, 1968; Weisstein, 1971)—are not consistent with the whole pattern of our results. Another possibility is that both “rapid” and “slow” processings contribute to the contextual cue processing in a different way. Considering that COCe processing at the early level is probably quite rapid (Blakeslee and McCourt, 2008), the visual system requires some additional mechanism to hold information about brightness estimation and information about contextual cues for the binding. The net COCe would be processed by co-operation of “rapid” and “slow” mechanisms as suggested in other brightness phenomena (Kaneko and Murakami, 2012; Cicchini and Spillmann, 2013). If this is the case, the size function becomes the sum of the temporally symmetric modulation function reflecting “rapid” processing and the temporally asymmetric function reflecting “slow” processing (Figure 10B). Further, the presence of additional slow mechanism might explain why the observed temporal characteristics of the COCe are sometimes slow (e.g., Davey et al., 1998). The breakdown of slow processing in a temporally high-rate display would cause a severe loss of contextual modulation and the observed COCe. In any case, however, drawing a conclusion about this problem is beyond the scope of the present study and will require further detailed examination.

In summary, the present results indicate that the contextual cue effect can be, at least partially, separated from the basic COCe processing. The visual system can bind information from brightness estimation by low-level mechanisms and contextual cues to modulate surface brightness. This indicates that the visual system has an additional mechanism to hold information about brightness estimation and information about contextual cues for the binding. This mechanism should be located after or at the same level as the binocular fusion but not necessarily at the higher and cognitive level.

### Conflict of interest statement

The authors declare that the research was conducted in the absence of any commercial or financial relationships that could be construed as a potential conflict of interest.
